# Protamine loops DNA in multiple steps

**DOI:** 10.1093/nar/gkaa365

**Published:** 2020-05-11

**Authors:** Obinna A Ukogu, Adam D Smith, Luka M Devenica, Hilary Bediako, Ryan B McMillan, Yuxing Ma, Ashwin Balaji, Robert D Schwab, Shahzad Anwar, Moumita Dasgupta, Ashley R Carter

**Affiliations:** Department of Physics, Amherst College, Amherst, MA 01002, USA

## Abstract

Protamine proteins dramatically condense DNA in sperm to almost crystalline packing levels. Here, we measure the first step in the *in vitro* pathway, the folding of DNA into a single loop. Current models for DNA loop formation are one-step, all-or-nothing models with a looped state and an unlooped state. However, when we use a Tethered Particle Motion (TPM) assay to measure the dynamic, real-time looping of DNA by protamine, we observe the presence of multiple folded states that are long-lived (∼100 s) and reversible. In addition, we measure folding on DNA molecules that are too short to form loops. This suggests that protamine is using a multi-step process to loop the DNA rather than a one-step process. To visualize the DNA structures, we used an Atomic Force Microscopy (AFM) assay. We see that some folded DNA molecules are loops with a ∼10-nm radius and some of the folded molecules are partial loops—c-shapes or s-shapes—that have a radius of curvature of ∼10 nm. Further analysis of these structures suggest that protamine is bending the DNA to achieve this curvature rather than increasing the flexibility of the DNA. We therefore conclude that protamine loops DNA in multiple steps, bending it into a loop.

## INTRODUCTION

During spermatogenesis, nuclear DNA is dramatically reorganized. In mammalian sperm, the DNA is compacted by a factor of ∼40 (1,2), with DNA condensation at almost crystalline packing levels (3,4). This densely packed DNA creates a more hydrodynamic sperm head for efficient swimming (5) and helps protect the DNA from UV radiation and DNA damage (6–9). Here we are interested in the pathway and mechanism that creates this dense packing.

**Figure 1.**
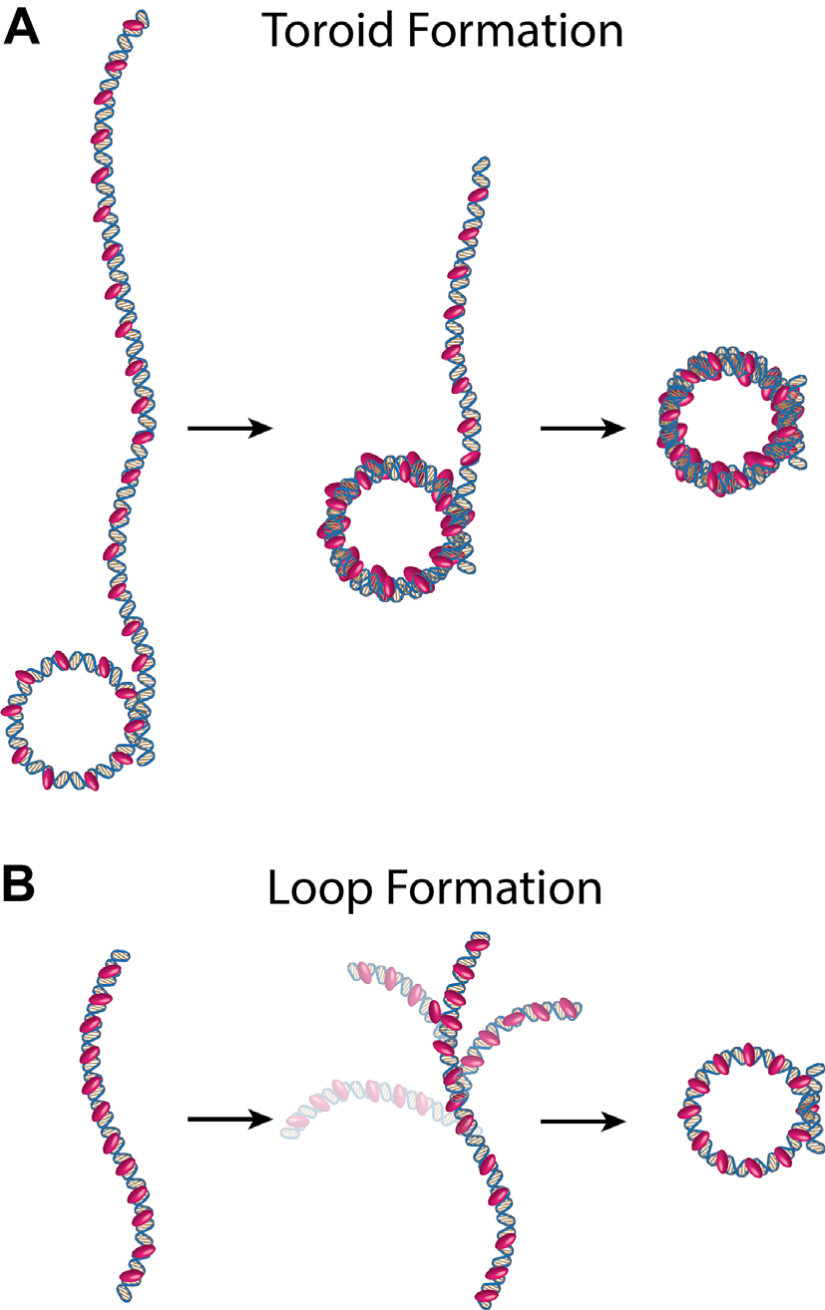
Models for toroid and loop formation. (**A**) A toroid formation model. Multiple protamine molecules (*pink*) bind the DNA and form a single loop. Subsequent loops stack one by one on top of the first loop, forming the toroid. (**B**) One-step loop formation model. Multiple protamine molecules bind the DNA. Random fluctuations in the conformation of the DNA then create opportunities for one or multiple protamine molecules to stabilize the location where the DNA strands cross, forming a loop in one step. Not to scale. Numbers of protamine molecules and binding locations could vary from what is shown.

At the heart of this process are protamine proteins. Protamines are small (∼50-amino-acid), arginine-rich proteins that have a highly positive charge (10,11) and are thought to bind in the groove of the DNA (2). *In vivo*, protamines are likely phosphorylated (12), but *in vitro* DNA condensation studies often use unphosphorylated protamines (2,13). To condense DNA, many of these positively charged protamines bind nonspecifically to the DNA, perhaps every ∼11 bp or so (2,14), neutralizing the negatively charged phosphate backbone (5,10,15). When there is enough protamine (>1 μM), the DNA begins to condense at a rate of ∼600 molecules/μM-s (13). This condensation produces a toroid of DNA (2,5,16–19). DNA toroids typically have outer diameters of ∼100 nm (19) and inner diameters of 15–85 nm (2,18). About 60 kb of DNA can be stored in a single toroid and sperm cells can have up to 50,000 toroids (17). Interestingly, other positively charged molecules, including hexaammine-cobalt (III), spermine, and spermidine, have also been shown to form DNA toroids (19,20). While the exact mechanism for the formation of a DNA toroid is unknown, measurements of DNA toroid condensation and decondensation by molecules other than protamine (21,22) show folding and unfolding of single loops from the toroid. Thus, the dominant model for toroid formation is that DNA is folded into a toroid loop-by-loop (Figure 1A), and that the first step in the pathway is the creation of a single loop of DNA (18,23,24). To begin characterizing toroid formation and packing by protamine, we therefore investigate the process for protamine to fold the DNA into a single loop.

One possible model for protamine to fold DNA into a single loop is a one-step, all-or-nothing model of looping. This one-step looping model has been proposed for bacterial transcription factors (25,26), like *lac* repressor (27–29) to loop DNA. In this one-step looping model, a single protein binds a single location on the DNA. Random fluctuations in the DNA then create opportunities for the protein to bind a second location, forming a loop. In the case of protamine, the one-step looping model is slightly different since multiple molecules of protamine would bind the DNA rather than a single molecule of protamine (Figure 1B). Still, the thought is that random fluctuations in the DNA would cause the DNA to flop over onto itself, creating an opportunity for one or multiple protamines to stabilize the location where the DNA strands cross (18,23). In this way, looping would occur in a single step.

To directly probe the DNA looping pathway, we used Tethered Particle Motion (TPM) to measure the real-time DNA folding dynamics (30). We expected looping by protamine to be a one-step process, with an unlooped state and a looped state. However, when we titrated small concentrations (0.1–0.4 μM) of protamine into the sample chamber, we saw multiple, long-lived (∼100 s) folded states. We also saw folding of DNA molecules that were too short to form loops. To visualize the DNA structures of these folded states, we used an Atomic Force Microscopy (AFM) assay (31). At low protamine concentrations (0.2–0.6 μM), we observed DNA molecules that were folded into loops and partially looped structures—c-shapes and s-shapes. We therefore propose a new multi-step looping model.

## MATERIALS AND METHODS

### Protamine and DNA constructs

We purchased salmon protamine (P4005, 1.1 kDa, Sigma Aldrich) which contained 32 residues and had a +21 charge (21 arginine residues). We dissolved the protamine and stored aliquots at -20°C.

DNA of varying lengths [*L* = 25 nm (75 bp), 50 nm (150 bp), 105 nm (309 bp) and 217 nm (639 bp)] was made via Polymerase Chain Reaction (PCR) using standard protocols (29,32–34), lambda phage DNA (New England Biolabs, N3011L) as a template, and LA Taq DNA polymerase (TaKaRa Bio, RR002). To biochemically tether the DNA to the surface of the cover slip and the streptavidin-coated polystyrene bead, one primer was labelled with a digoxigenin molecule, while the other was labelled with a biotin molecule (Integrated DNA Technologies). To purify the DNA, we performed gel electrophoresis using orange loading dye (B7022S, New England Biolabs) and standard protocols for short DNA molecules (35), then purified the DNA via gel extraction (QIAquick Gel Extraction Kit, Qiagen). We quantified the final concentration using a spectrophotometer (Nanodrop One, ThermoFisher Scientific).

### Sample preparation for AFM assay

To create samples, we adhered mica slides (Ted Pella, grade V1 10-mm-diameter ruby muscovite mica) using epoxy or double-sided scotch tape to 28-mm-diameter, magnetic, stainless steel disks. Then, we cleaved the mica to expose a clean layer by pressing tape onto the surface of the coverslip and quickly removing. We made control slides by adding a solution of 1 ng/μl DNA and 2 mM magnesium acetate to the slide, incubating for 2 s, washing with 1 ml deionized water, and blowing the sample dry using nitrogen. To prepare samples with protamine, we used solutions with a more dilute DNA concentration (0.1–0.4 ng/μl) to prevent molecular clumping and added 0.2–2.0 μM protamine and 2 mM magnesium acetate. Then, we applied the solution to the slide as before, but used 2–5 depositions to increase the number of molecules attached to the surface. Specifically, we used 0.4 ng/μl of 398-nm- or 217-nm-length DNA (2 depositions), 0.2 ng/μl of 105-nm-length DNA (five depositions) and 0.1 ng/μl for 50-nm-length DNA (five depositions). Samples were stored in a desiccator.

### AFM assay data collection and analysis

We used a Dimension 3000 AFM (Digital Instruments) with a Nanoscope IIIa controller to image samples and PPP-XYNCSTR-model cantilevers (NanoSensors, resonant frequency = 150 kHz, force constant = 7.4 N/m, length = 150 μm, tip radius < 7 nm) (Supplementary Figure S1A). All images were taken in tapping mode. The scan rate ranged from 1–6 Hz. Images are 1 μm × 1 μm and contain 256 × 256 pixels. Images were processed using Gwyddion (36) or SPIP. For all images, we aligned rows using a fifth degree polynomial.

To create a library of images of single DNA molecules (Supplementary Table S1), we selected single molecules using ImageJ v1.51 (37,38) that were at least 1 pixel away from other molecules and were contained within the field of view (Supplementary Figure S1B). To be selected, molecules also had to lay flat along the surface (having a contour length of 80% of the actual value) and have the expected height of ∼0.5 nm. We cropped a 200 nm × 200 nm image and thresholded it so that the DNA was white against a black background.

Thresholded images were analysed in MATLAB (MathWorks) (Supplementary Figure S1C and D). To calculate the end-to-end extension of a molecule, we determined the DNA endpoints by computing the curvature around the boundary and designating the endpoints as the locations of maximum curvature. In some cases, particularly in molecules that were looped, this procedure did not correctly determine the two endpoints. In that case, the user manually selected the endpoints. Finally, we computed the contour of the DNA by averaging the boundary on either side of the endpoints. The contour of the DNA is the longitudinal axis of the DNA.

We classified molecules using the following scheme. Molecules that had unfilled areas, *A*, were marked as loops. The diameter, *d*, of the loop was calculated by adding the diameter of the unfilled area, $2\sqrt {\frac{A}{\pi }}$, to the DNA molecule width (2.5 ± 0.5 pixels) in our thresholded images. Molecules that have extensions ≥60% of their contour length were marked as linear. Molecules that have extensions <60% of their contour length were marked as being in an intermediate folding state as they were folded but not looped. These molecules could be s-shape (the curvature switches sign), c-shape (the curvature has a single sign), or pseudo-loops (the DNA cross over itself, but does not have an unfilled area).

We used the ‘combo_calc’ function from the Easyworm analysis suite (39) in MATLAB to analyze the structures of the molecules. Specifically, Easyworm calculates the decay in the tangent-tangent correlations along the contour of the DNA molecule. The length along this contour is the contour length, *ℓ*. Easyworm also calculates the mean-squared displacement (MSD) along the contour. Finally, we calculated the radius of curvature *R* of the molecules using the ‘LineCurvature2D’ function (40).

### Sample preparation for TPM assay

To prepare samples for the TPM assay (Supplementary Figure S2), we first constructed a 15-μl-volume sample chamber that would allow for buffer exchange. This sample chamber was assembled using a cover slip (Fisher Scientific, 12-544-B), microscope slide (Corning, 2947), and double-sided sticky tape as a spacer, and was held together with 5-minute epoxy, similar to prior studies (41,42).

Next, we non-specifically bound antidigoxigenin to the cover slip (29,34,37,43) by incubating 20 μg/ml antidigoxigenin [from sheep, Roche, diluted in 100 mM sodium phosphate buffer (pH 7.5)] within the flow chamber for 60 min at room temperature. After incubation, the flow chamber was rinsed by flowing 400 μl of wash buffer (WB), [25 mM Tris–HCl (pH 7.5), 1 mM magnesium acetate, 1 mM sodium chloride, 1 mM dithiothreitol, 0.4% Tween-20 (a non-ionic detergent), and 3 mg/ml bovine serum albumin (BSA, a blocking agent)]. To make 3 mg/ml BSA solution we diluted 25 mg/ml BSA (Sigma Aldrich, A3059) in 0.4% Tween-20 and 20 mM Tris–HCl. Concentrations cited are those before filtration through a 0.2-μm filter.

Concurrent with the antidigoxigenin incubation, we prepared a bead-DNA mixture (30,32–34,44) with 170 pM streptavidin-coated, polystyrene beads (560-nm-diameter, Spherotech, SVP-05-10) and 100 pM DNA. Prior to mixing the DNA and beads, we washed the beads with 0.4% phos-tween [0.4% Tween-20 diluted in sodium-phosphate solution (pH 7.5)] and sonicated them in a cup sonicator (QSonica, Q500 with Oasis 180 chiller) for 60 minutes (2 s pulse on, 2 s pulse off) so they were monodispersed (33).

Finally, we added the bead-DNA mixture to the sample chamber and let it incubate overnight before washing. To titrate protamine into the assay, we used 0.1–0.4 μM of protamine dissolved in WB and flowed 200 μl of each concentration before recording. Flow was along the *y* direction of the microscope.

### TPM data collection and analysis

Sample chambers were imaged with a bright-field Nikon Eclipse Ti-U inverted microscope. This microscope used a 100×, oil immersion, objective lens (Nikon, CFI Plan Apochromat Lambda Series, numerical aperture = 1.45), and a 12-V, 100-W halogen lamp (Nikon, D-LH/LC Lamphouse). Images were recorded with a CoolSnap EZ camera (Photometrics, full field of view = 1392 × 1040 pixels, camera pixel size = 6.45 μm × 6.45 μm) at 5 Hz. Videos for control data without protamine were 1000–2000 frames, and videos with protamine were 2000–4000 frames. Image exposure time was 10 ms and the time between images was 200 ms. The camera settings were controlled by Micro-Manager 1.4.22 (45), an open-source image acquisition software.

Each series of images was imported into ImageJ v1.51 for analysis. To perform the analysis, the 16-bit images were converted to 8-bit and thresholded (46) to create binary images. Next, beads were tracked using MTrack2 software (47), which outputs the *x* and *y* positions for each bead tethered to the surface.

Data was analysed in IGOR Pro v6.37 (WaveMetrics). Specifically, we imported *x* and *y* particle positions for each tether, converted them from pixels into nanometers, and removed the low-frequency instrumental drift (48,49) by fitting a line to the data and subtracting that line (44,50). For control data without protamine, particle positions, *x* and *y*, were required to have an eccentricity $(e\ = \sqrt {| {1 - \frac{{_{{\sigma _{\rm{x}}}}}}{{{\sigma _{_{\rm{y}}}}}}} |} \ )$ <0.45. This cut on eccentricity ensures we only measure particles tethered to a single DNA molecule (32,44,50,51). A larger eccentricity indicates that the particle is tethered to more than one molecule. In addition, the standard deviation for the whole trace was required to be greater than a cut-off value (>18 nm for *L* = 25 nm, >25 nm for *L* = 50 nm, and >40 nm for *L* = 105 nm). This selection ensured that particles were bound to DNA molecules of the correct length.

To analyze DNA folding, we used several metrics. First, we measured the rolling standard deviation of the particle, *σ*_x_, by calculating the standard deviation of a trace over a particular window (50 points) and then advancing or rolling the window over by one point and recalculating the standard deviation for this new window. Continued advancement of the window creates a time-dependent, rolling standard deviation at 0.1 Hz. If the DNA molecule folds, this rolling standard deviation decreases. Second, we used histograms of *σ*_x_ to determine the states of the DNA molecule. We histogram the rolling, standard deviation trace at 0.1 Hz using a bin size of 1 nm. We identify peaks in the *σ*_x_ histogram (*σ*_x_ peaks) using an algorithm that requires the selected bin to be taller than the adjacent bins and have a count greater than the size of the sliding window (50 points). A transition in a trace occurs if the *σ*_x_ values in the trace move from one peak in the histogram of *σ*_x_ to another peak. To pool all of the data from all of the molecules of count *N* together, we also plot a *σ*_x_ peaks histogram with a bin size of 3 nm (set by the noise level of the control traces in Supplementary Figure S2). Control traces of particles nonspecifically stuck to the surface with 5 mM magnesium acetate have a standard deviation of 3 ± 1 nm. Thus, we conservatively set the detection limit of the instrument to the end of the second bin, which is at 6 nm.

## RESULTS

### Protamine folds DNA into single loops

Models of toroid formation (18,21–23,52) assume that protamine and other condensing agents can form single loops of DNA. Some models also propose that the nucleation event for the toroid is the first loop of DNA that forms (18,23,52). Here we sought to visualize these single DNA loops.

**Figure 2.**
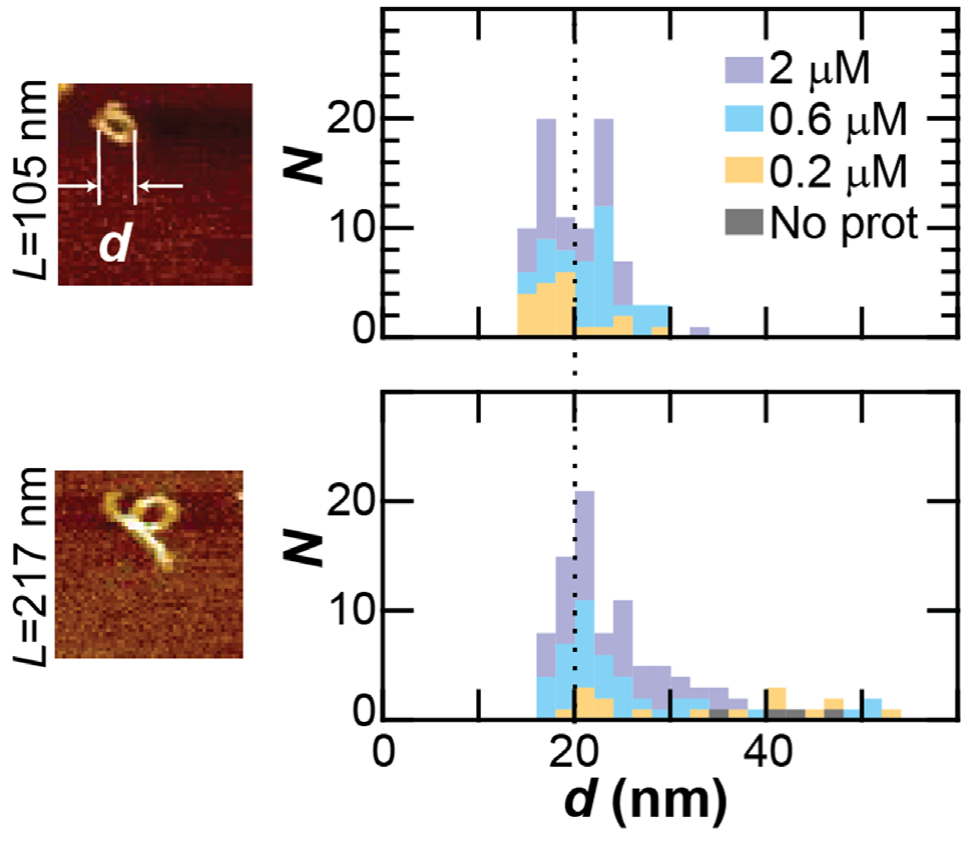
DNA loops folded by protamine have ∼20 nm diameters. (Left) Images of DNA loops folded by protamine. DNA length, *L*, is either 105 nm or 217 nm. Computer algorithm calculates the loop diameter *d* (see Materials and Methods). Width of images is 200 nm. (Right) Histograms of the loop diameter at different protamine concentrations (*gray* = no protamine, *yellow* = 0.2 μM, *blue* = 0.6 μM, and *purple* = 2 μM protamine). Histograms are stacked. Mean loop diameter for 105-nm-length DNA is 20 nm (*dotted line*).

To visualize loops of DNA folded by protamine, we fixed the DNA (*L* = 105 nm or 217 nm) to a mica slide without protamine, and at both low (0.2 and 0.6 μM) and high (2 μM) protamine concentrations. High protamine concentrations of >1 μM cause complete condensation of large, 50-μm-length DNA (13). After the DNA was fixed to the sample surface, we scanned an AFM tip over the sample to record an image of the DNA topology. In scans without protamine, we observed linear DNA molecules on the surface. However, when we added protamine, we found that the DNA folded and clumped together. Since clumping is between molecules and is most likely due to protamine interactions that facilitate toroid formation, we did not study clumping further. Instead, we searched the AFM scans for individual molecules that were looped. Sure enough, we were able to form a library of such molecules at both DNA lengths (Figure 2) and all of the protamine concentrations tested (numbers of molecules at each concentration and DNA length located in Supplementary Table S1). For 217-nm-length DNA, we also found molecules that formed toroids with multiple loops, and so we limited our library to molecules with only a single loop. In addition, 217-nm-length DNA formed spontaneous loops in the absence of protamine, which did not occur in the 105-nm-length DNA. These spontaneous loops are due to the random fluctuations of the longer DNA molecule as it lays onto the sample surface.

Given this data, we conclude that protamine can loop DNA. Our goal now is to elucidate the pathway and dynamics for how this loop forms.

### Protamine has a preferred loop diameter

After observing single DNA loops, we next sought to characterize the diameter, *d*, of these loops. One might expect a wide-range of diameters (tens to hundreds of nanometers) if protamine uses a one-step looping strategy (23) that requires waiting for spontaneous DNA folding events. When we measure spontaneous looping in 1023-nm-length DNA molecules we measure loop sizes of 14–150 nm (*data not shown*). However, if protamine uses a different strategy, then there might be a preferred diameter for the first folded loop of DNA. Would the loops show a wide-range of diameters or a single preferred diameter?

To measure the diameter of our DNA loops, we used a computer algorithm to extract the diameter from each image that corresponded to a molecule with a single loop (Supplementary Figure S1). For 105-nm-length DNA, a histogram of the loop diameter shows a clump around a diameter of ∼20 nm (Figure 2). The measured diameter is 19 ± 4 nm (mean ± standard deviation), 22 ± 4 nm, and 20 ± 4 nm at 0.2, 0.6 or 2.0 μM protamine, respectively. The measured loop diameter for loops at all concentrations is 20 ± 4 nm. In addition, at all of the concentrations measured, the data is clustered between loop diameters of 14–35 nm. The smallest diameter of 14 nm corresponds to the smallest loop that we can measure as this loop encloses 1 pixel. Loops that do not enclose at least 1 pixel are classified as pseudo-loops (Supplementary Figure S3). The largest diameter of 35 nm corresponds to the entire 105-nm-length molecule folded into a loop. Thus, we see the full range of possible loop diameters with protamine, though we never see any spontaneous loops forming without protamine.

When we measure the loop diameter for the 217-nm-length DNA, we do not see the full range of possible loop diameters. For the 217-nm-length DNA, the histogram of loop diameter is peaked at 20 nm, and most of the data lies in the 14–35 nm range, similar to the 105-nm-length DNA data. Still, there is a longer tail on the histogram at higher diameters, which shifts the mean loop diameter for all concentrations to 26 ± 8 nm. This tail extends all the way to a diameter of 60 nm and is probably due to spontaneous DNA loop formation. Spontaneous loops are present on 217-nm-length DNA without protamine and have larger diameters of 35–60 nm.

A measured loop diameter of ∼20 nm is interesting for several reasons. First, it is much smaller than the toroid outer diameter of 100 nm (19) and is comparable to the smallest measurements of the toroid inner diameter (2,18). If toroids do form using a nucleation loop, then our data would suggest that the nucleation loop is one of the smallest loops in the toroid, perhaps allowing the toroid to expand outward as it grows. Second, it is not likely that the small-diameter loops we observe are created by stabilizing spontaneous loops that form naturally in naked DNA. The smallest spontaneous DNA loops we measure for 217-nm-length DNA are 35 nm in diameter, larger than the protamine-induced loops. However, we do note that *lac* repressor, which is thought to stabilize spontaneous DNA loops, has been previously reported to loop 4 turns of the DNA helix (40 bp or 14 nm) (53). Finally, the histogram of loop diameter is peaked at ∼20 nm, indicating a preferred diameter for protamine folding, contrary to our expectation of wide-ranging loop diameters. To gather more information about the pathway for DNA looping, we decided to measure DNA looping in real time.

### Real-time folding shows multiple, long-lived, reversible states

If DNA looping by protamine follows the same one-step looping strategy as bacterial transcription factors (29), which wait for spontaneous DNA fluctuations to form a loop, then we expect our real-time measurements to have a single DNA folding event. This would be folding from an unlooped state to a looped state. Previous *in vitro* real-time measurements (21,22,54) corroborate this expectation, as DNA folding or unfolding from toroids (created by other condensing agents) shows discrete steps in DNA length that are thought to correspond to single loops.

**Figure 3.**
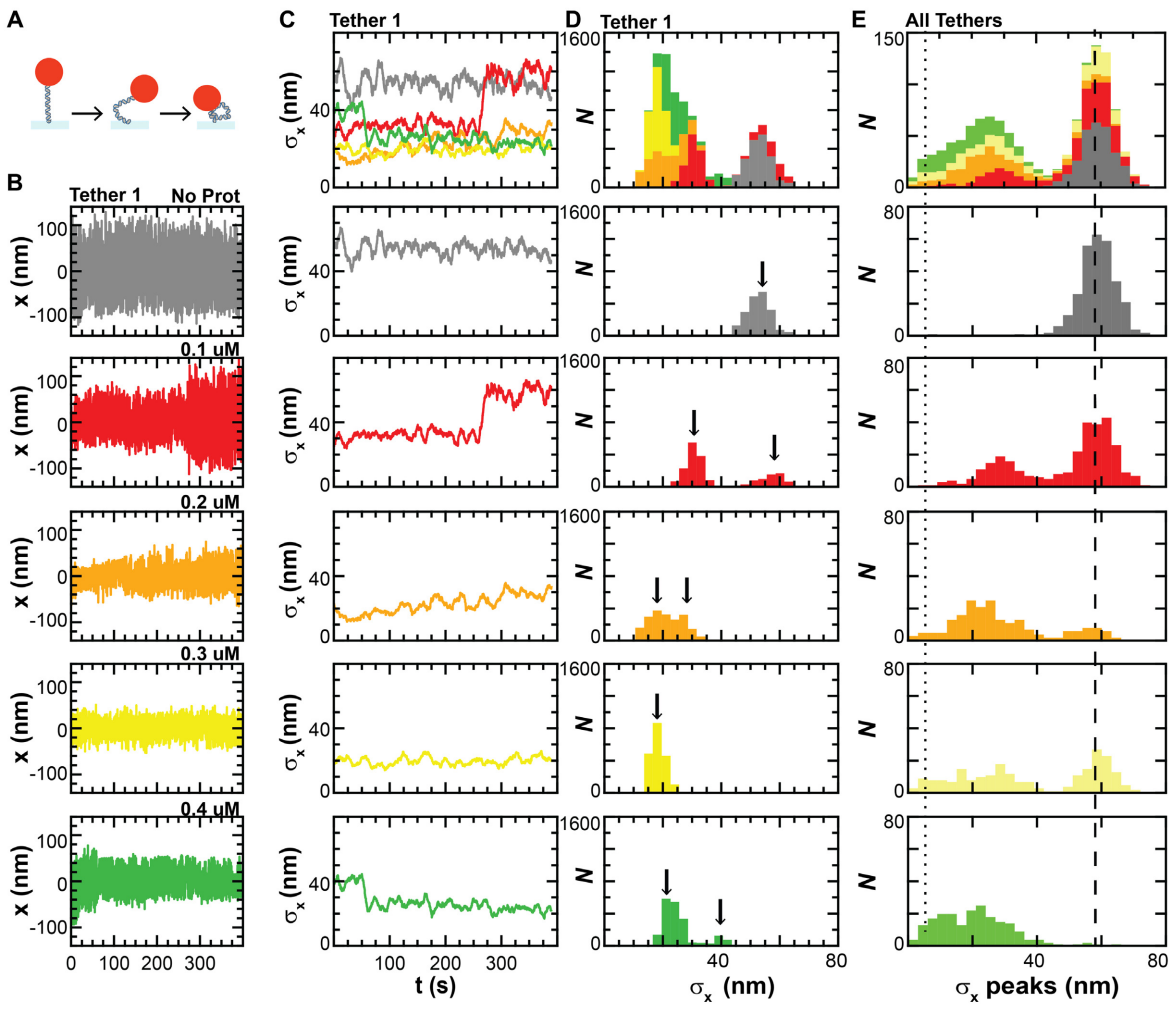
Loop formation is multi-step with long-lived, reversible states. (**A**) Assay consists of particle tethered to the surface via a DNA molecule. As protamine loops the DNA, the tether shortens, decreasing particle motion. (**B**) Particle position, *x*, at 5 Hz for a single tether without protamine (*gray*) and with protamine titrations of 0.1 μM (*red*), 0.2 μM (*orange*), 0.3 μM (*yellow*), and 0.4 μM (*green*). (**C**) Rolling standard deviation, *σ*_x_, at 0.1 Hz of the *x* traces at each concentration plotted together (*top*) and individually (*bottom*). Traces of *σ*_x_ decrease as the protamine concentration increases and the DNA folds. (**D**) Histograms of the *σ*_x_ traces at each concentration stacked together (*top*) and plotted individually (*bottom*) show the presence of multiple peaks (*black arrows*), indicating multiple folded states. The height of the peak is equal to the number of frames in the state, with many states lasting longer than 100 s. (**E**) We repeat the measurement made in B-D on all the tethers (*N* = 103) and determine the peaks in the *σ*_x_ histogram (*σ*_x_ peaks). We then histogram the *σ*_x_ peaks for all tethers at each concentration, stacking them together (*top*) and plotting individually (*bottom*). The unfolded state has a mean *σ*_x_ of 59 ± 5 nm (*dashed line*). The detection limit (*dotted line*) of the instrument is 6 nm.

To measure the real-time looping of DNA, we used a single-molecule, TPM assay (Figure 3A). Single molecule assays are useful for measuring dynamics as they do not require molecular synchronization (55). In the TPM assay, we attached a polystyrene particle to a single DNA molecule (*L* = 105 nm) and tethered it to the surface. We then titrated in different concentrations of protamine (0.1–0.4 μM) and watched the motion of the tethered particle decrease as the protamine folded the single DNA molecule (Figure 3B). If protamine loops the DNA in a single step, we should see only one unfolded state and one folded state. Instead, as protamine was titrated into the sample, we observed that the DNA molecule entered multiple, long-lived (>100 s) states that corresponded to different standard deviations of the particle, *σ*_x_ (Figure 3C). When we plotted a histogram of these *σ*_x_ values (Figure 3D), we saw five different peaks for this particular molecule: the unfolded state and 4 folded states. In looking at other individual tethers (examples of 6 other tethers in Supplementary Figure S4), we observed similar features with 2–4, long-lived, folded states.

To quantify the folding for all the tethers (*N* = 103 molecules), we histogrammed the peak locations in the *σ*_x_ histogram (*σ*_x_ peaks) at each protamine concentration (Figure 3E). Without protamine, the DNA tethers all exist in a single, unfolded state, clustered at a mean *σ*_x_ of 59 ± 6 nm (fractional *σ*_x_ = 1). When we add 0.1–0.2 μM protamine, however, the DNA tethers begin to fold, adopting decreased *σ*_x_ values, many between 20–35 nm (fractional *σ*_x_ = 0.3–0.6). If we increase the protamine concentration even further to 0.4 μM, then 100% of molecules have folded below <51 nm and 45% of *σ*_x_ peaks are between 6 and 21 nm.

In addition to observing multiple folded states, we also noticed that DNA folding by protamine was reversible. Specifically, we observed increases in *σ*_x_, indicating a reverse in the DNA folding. To quantify this reversibility, we first identified transitions between two states. A transition is defined as a change in the particle's standard deviation, *σ*_x_, from one peak in the *σ*_x_ histogram (the start location) to another peak (the end location). Some DNA molecules (*N* = 34) fold or unfold during buffer exchange when protamine is being titrated into the sample chamber (e.g. Supplementary Figure S4, Tether 6), other molecules (*N* = 69) show forward (e.g. Figure 3, *green*) or reverse (e.g. Figure 3, *red*) transitions. When we compiled these transitions together (Supplementary Figure S5), we noticed that all binned values of *σ*_x_ were both start locations and end locations, indicating that reverse transitions can occur at all of the folded *σ*_x_ values. Thus, we conclude that the multiple, long-lived, folded states we observe are also reversible.

Interestingly, only half of the reverse transitions are discrete (≤2 s), with some having transition times >10 s (Supplementary Figure S5). For comparison, 80% of the forward transitions are discrete. These two different transition times could indicate the presence of competing folding pathways—one discrete and one gradual. But, more likely, the two different transition times indicate that our spatio-temporal resolution is limiting our ability to distinguish states with either small (<3 nm) *σ*_x_ changes or fast (≤2 s) transition times. If the latter is true, then there are likely to be even more states in the DNA looping pathway than the 2–4 folded states we observe.

Finally, we note that the data does not describe the nature of the folded states we observe. One hypothesis is that these folded states all correspond to different single-loop states. Previous TPM data has reported that *lac* repressor has more than one single-loop state (28,29). Another hypothesis is that protamine is folding the DNA into intermediate states in the looping pathway. Folding the DNA requires a decrease in the Gibbs free energy, *G*, which is related to the enthalpy, *H*, the temperature, *T*, and the entropy, *S*, of the system by the relation:

(1) $$\begin{equation*}\Delta {G} = \Delta {H} - {T}\Delta {S}.\end{equation*}$$

Since the temperature in the experiment is constant, folding could be due to a decrease in the enthalpy (*e.g*. a bend in the DNA) or an increase in the entropy (e.g. an increase in the flexibility of the DNA). Both changes (bending or flexibility increases) would appear as decreases in the standard deviation in the TPM assay. As more protamines bind the DNA at the higher protamine concentrations, we might expect different amounts of bending or DNA flexibility, causing the various folded states. We will thus need a test that can determine whether these folded states are different single-loop states or intermediate folding states.

### Folded states form even without looping

To test whether the folded states we measure with our TPM assay are single-loop states or intermediate folding states, we looked to see if DNA molecules shorter than the circumference of a loop would fold. If these short molecules fold, then protamine is folding the DNA into intermediate folding states other than a loop.

**Figure 4.**
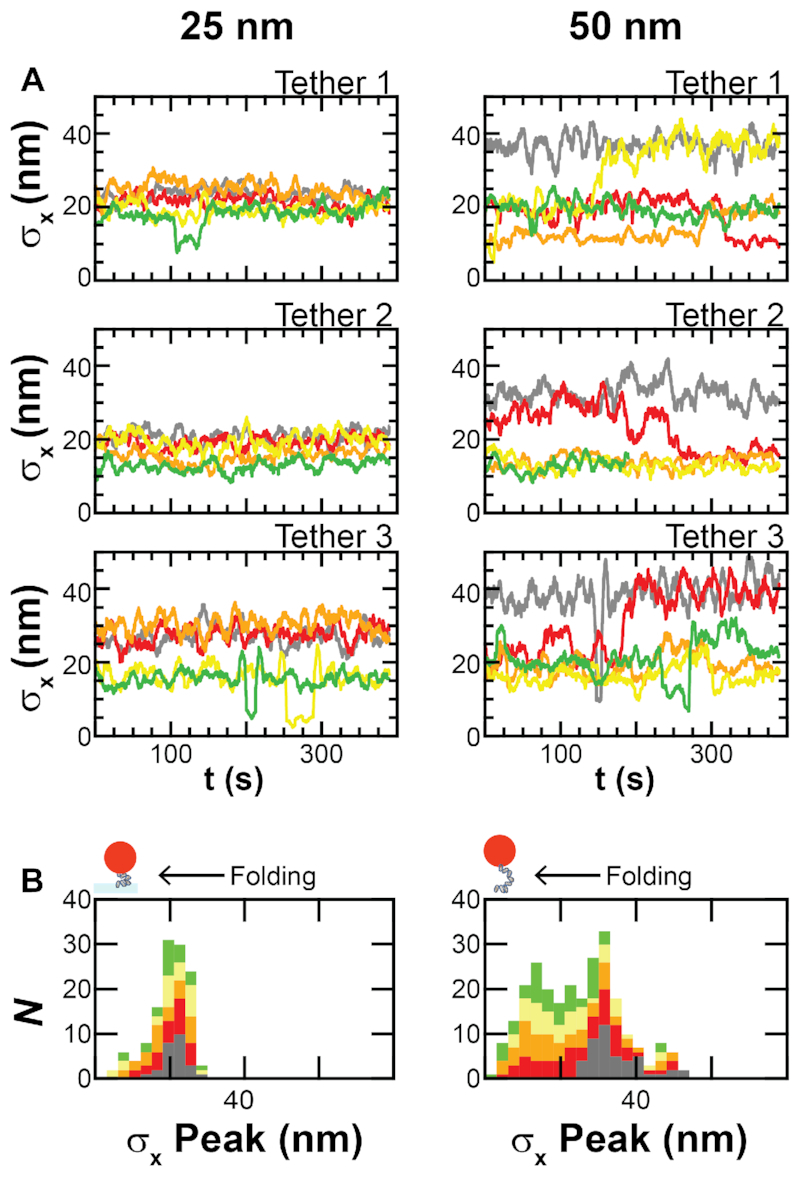
DNA folding occurs even at short DNA lengths. (**A**) Plots of the rolling standard deviation, *σ*_x_, at each protamine concentration over time for three individual 25-nm-length DNA tethers and three individual 50-nm-length DNA tethers. A decrease in *σ*_x_ indicates DNA folding. Data is at 0.1 Hz. (**B**) Histograms of *σ*_x_ peaks for all of the 25-nm-length and 50-nm-length DNA tethers. Histograms at each protamine concentration are stacked one on top of the other. Color scheme is the same as Figure 2.

To test for folding of short DNA molecules, we used our TPM assay to measure the folding dynamics of two different lengths of DNA: 50 nm and 25 nm (*N* = 27 molecules for *L* = 50 nm, and *N* = 16 molecules for *L* = 25 nm). DNA molecules that are ≤50 nm (150 bp) do not form loops in the AFM assay (Supplementary Figure S6). DNA molecules that are 25 nm (75 bp) long have seven turns of the DNA helix and therefore can be bound by up to seven protamine molecules (14,16). We titrated protamine at 0.1–0.4 μM into our samples and measured the standard deviation of the particle motion for DNA tethers at each length and concentration (Figure 4). We were able to observe instances of folding for both of these short DNA molecules. Histograms of *σ*_x_ peaks for the 50-nm-length DNA depicted 80% of tethers changing from the unfolded state at a mean *σ*_x_ = 35 ± 7 nm in the absence of protamine to a folded state at a *σ*_x_ <24 nm in the presence of protamine. In addition, *σ*_x_ traces of individual 50-nm-length DNA tethers showed decreases from the unfolded state to 1–3 folded states that were long-lived (>100 s) ( Figure 4), and reversible (Supplementary Figure S5), analogous to the states measured for the 105-nm-length DNA molecules. Similarly, histograms of *σ*_x_ peaks for the the 25-nm-length DNA tethers showed some (25%) movement from the unfolded state at a mean *σ*_x_ = 21 ± 3 nm in the absence of protamine to a folded state at *σ*_x_ of 6–15 nm in the presence of protamine. These folded states were above the detection limit of the instrument (6 nm). Traces of *σ*_x_ for individual 25-nm-length DNA tethers showed 0–2 folded states that were long-lived (>100 s) and reversible.

**Figure 5.**
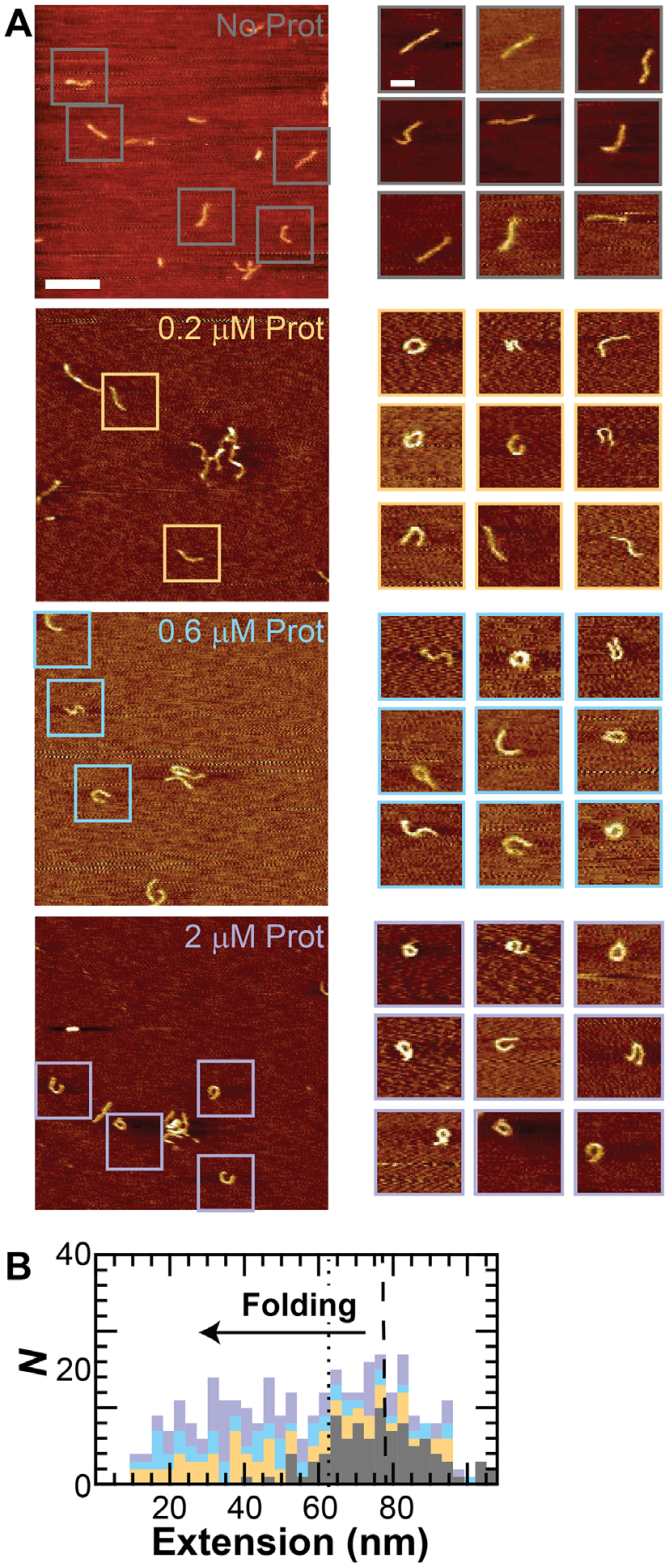
Intermediate structures are partial loops. (**A**) (*Left*) AFM images of DNA (*L* = 105 nm) without protamine (*gray*) and with 0.2 μM (*orange*), 0.6 μM (*blue*), or 2 μM (*purple*) protamine show individual molecules (*box*). Scale bar is 200 nm. (*Right*) Representative individual molecules show unfolded and folded structures. The folded structures are loops and partial loops—c-shapes and s-shapes. Scale bar is 50 nm. (**B**) Computer algorithm calculates the end-to-end extension (*inset*) for all molecules that are not loops. Plot shows stacked histograms of the extension. Color scheme same as in A. Unfolded molecules have a mean extension of 77 nm (*dashed line*). Molecules that are in an intermediate folding state are defined as having a fractional extension of <0.6 (*dotted line*).

Given these observations, we conclude that protamine is able to fold short (25–50 nm) DNA molecules with the same long-lived, reversible mechanism that it uses for folding 105-nm-length DNA molecules. This suggests that the folded states we observe are not different single-loop states, but are rather intermediate folding states. The presence of these intermediate folding states indicate that protamine is using a multi-step looping model.

### Intermediate folding states are partial loops

After discovering these intermediate folding states, we sought to image their structures. We used our AFM assay to measure the topology of 105-nm-length DNA molecules affixed to the surface in varying protamine concentrations (0, 0.2, 0.6 and 2 μM). We then searched the AFM scans for individual molecules that were within 80% of the correct DNA contour length and did not contain any loops (numbers of molecules listed in Supplementary Table S1). A computer algorithm (Supplementary Figure S1) extracted the DNA end-to-end extension for all of the non-looped molecules. We use end-to-end extension as the proxy for DNA folding since it is similar to the tether length measurement in the TPM assay. Without protamine (Figure 5), the DNA is unfolded at a mean extension of 77 ± 15 nm, and only 15% of the molecules had extensions <63 nm (fractional extension <0.6). With protamine, however, 69% of the molecules fold to extensions <63 nm. We define these DNA molecules with fractional extensions <0.6 as being in the intermediate folding state. We then looked to see if there were any defining characteristics for the DNA structures in this state. Qualitatively, the structures all appear to be partial loops—structures with the curvature of a loop but without the full enclosure of a loop. Specifically, these partial loops are c-shapes (78%), s-shapes (20%) or pseudo-loops (2%) (Supplementary Figure S3).

We repeated the procedure for 50-nm-length DNA molecules (Supplementary Figure S6). Without protamine, 50-nm-length DNA molecules appeared as unfolded, linear molecules with a mean extension of 45 ± 7 nm. Only 3% of these molecules had fractional extensions <0.6. However, when we added 0.2–2 μM protamine, the DNA folded to a mean extension of 35 ± 9 nm, and 27% of molecules had fractional extensions <0.6. Structures for the molecules in the intermediate folding state were again partial loops—either c-shapes (96%) or s-shapes (4%).

### Intermediate folding states have a radius of curvature of ∼10 nm

Finally, our goal was to determine the physical mechanism that protamine uses to fold the DNA. Is it changing the enthalpy or the entropy of the DNA?

To distinguish between these possibilities, we can measure the persistence length, *L_p_*, and radius of curvature, *R*, for molecules with intermediate folding states. Mathematically, the persistence length is the length over which the tangent vector to a polymer remains correlated or persists. Physically, the persistence length is a measure of the flexibility of the polymer, since a decrease in the persistence length is indicative of a more flexible polymer. A naked DNA molecule has a nominal persistence length of 50 nm (56,57). If we measure the structure of molecules in intermediate folding states to have a lower persistence length, then it is likely that the DNA is folding entropically. If, on the other hand, we measure the molecules in the intermediate folding states to all have the same radius of curvature, then the DNA is folding enthalpically due to a change in the bend of the molecule. This radius of curvature should match our measurements of loop radius at ∼10 nm.

**Figure 6.**
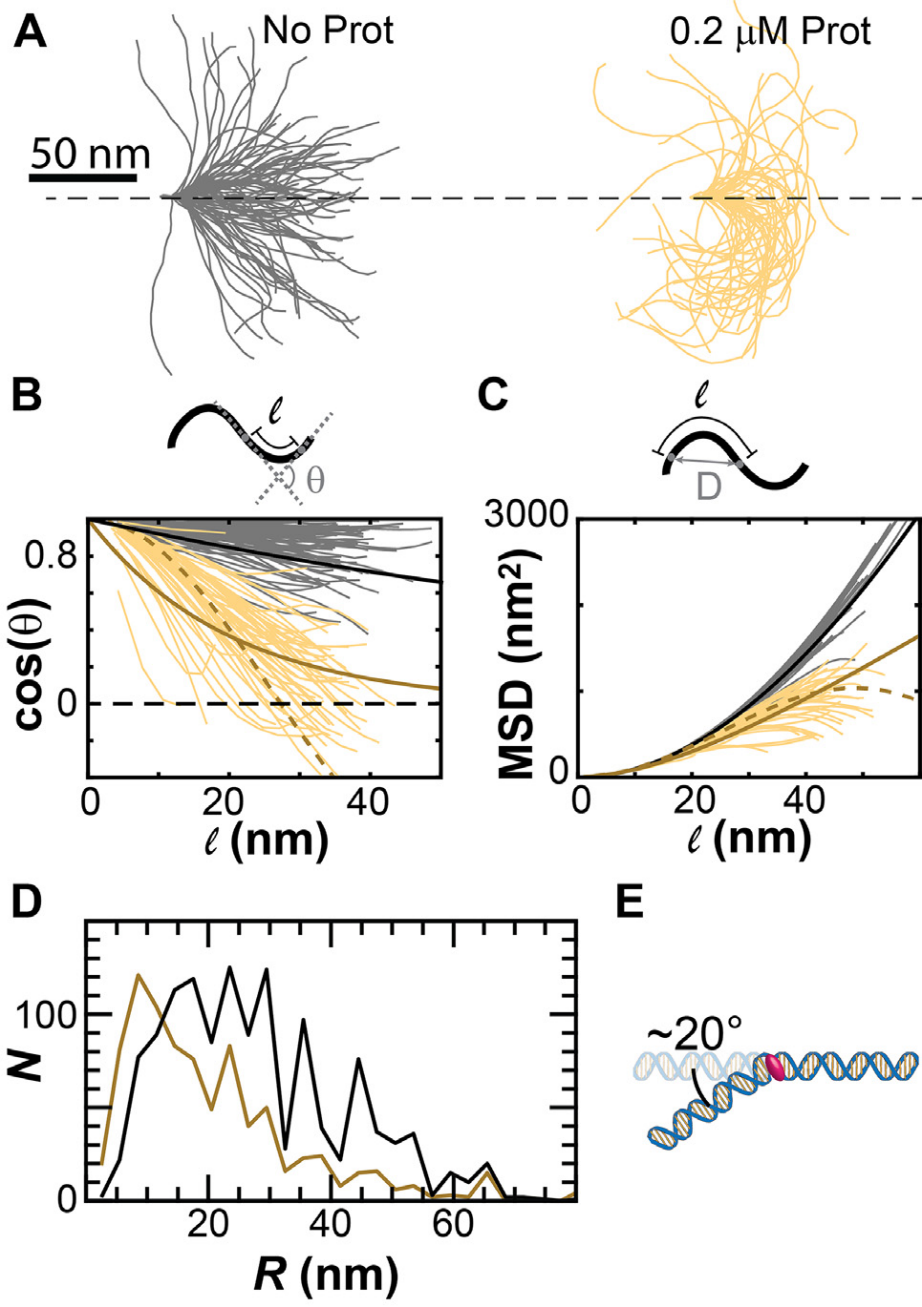
Intermediate folding states have a radius of curvature of ∼10 nm. We compare DNA molecules without protamine (*gray*) to molecules with 0.2 μM protamine that are folded but not looped (*yellow*). (**A**) Contours for the molecules are aligned so that they start at the origin and initially have tangent vectors that point along the *x*-axis. (**B**) The cosine of the angle, *θ*, between segments along the contour decreases with contour length, *ℓ*. Fits using the model of the flexible polymer (*bold, solid lines*) approach zero. The fit using the model of the contour bending along a circle (*dashed line*) crosses over zero and becomes negative. (**C**) The mean-squared displacement (MSD) calculated from the displacement *D* for points along the DNA contour as a function of contour length. Fits using the model of the flexible polymer (*bold, solid lines*) increase with contour length. The fit using the model of the contour bending along a circle (*dashed line*) decreases at high (>40 nm) contour lengths. (**D**) Histograms of the radius of curvature for molecules in 0.2 μM protamine that are folded but not looped (*dark yellow*) and molecules without protamine (*black*). (**E**) Cartoon of single protamine creating a 20° bend in the DNA.

Here we measured the persistence length of the DNA (*L* = 105 nm) for (i) linear molecules without protamine and (ii) molecules that were in the intermediate folding state at 0.2 μM protamine using three methods. We first plot the contours of all of the DNA molecules tested (Figure 6A), arranging them so that they all begin at the origin and have their tangent vector parallel to the *x*-axis. Without protamine, the DNA molecules appear straight over the 100 nm, with 60% lying along the *x*-axis to within 40 nm. However, with protamine, the DNA molecules in the intermediate state start to bend away from the x-axis at the 15–40 nm mark and wrap back toward the origin. The endpoint for 90% of these molecules is ∼30 nm from the origin. While a decrease in persistence length would account for the lack of straight molecules, it would not account for all of the molecules with the intermediate folding structure to follow the same path.

In the second method (Figure 6B), we plot the decay of the tangent-tangent correlations (39) along the contour of the DNA. A flexible polymer with a particular persistence length should follow the equation:

(2) $$\begin{equation*}\left\langle {{\rm{cos}}\,\theta } \right\rangle = {e^{\frac{{ - \ell }}{{s{L_p}}}}}.\end{equation*}$$

In this equation, *θ* is the angle between the tangent vectors, *ℓ* is the independent variable which is the length along the contour, and *s* is the dimension of the data (*s* = 2 for 2D data). A curve with a persistence length of 60 nm captures the behaviour of the linear DNA molecules without protamine, as these should be flexible polymers. However, the fit for the molecules in the intermediate folded state (*L*_p_ = 10 nm) deviates from the data at both low (<10 nm) and high (>40 nm) contour lengths. At contour lengths >30 nm, the data crosses the *x*-axis, indicating a negative correlation, while the fit falls off to zero. These molecules in the intermediate folding state do not appear to be flexible polymers with a decreased persistence length. If we instead assume that the DNA is bent into a loop, then we would expect the data to follow the tangent-tangent correlation for a circle. A tangent-tangent correlation for a circle with radius of 18 nm does fit the data. This curve captures both the negative correlation >30 nm and the change in the data from concave down to concave up

The third method (Figure 6C) for obtaining the persistence length is to measure the mean squared displacement (MSD) for points along the contour as the length along the contour increases (39). A flexible polymer with a given persistence length should have an MSD that increases as the contour length increases:

(3) $$\begin{equation*}{\rm{MSD}}(\ell ) = 2s{L_p}\ell \left( {1 - \frac{{s{L_p}}}{\ell }\left( {1 - {e^{\frac{{ - \ell }}{{s{L_p}}}}}} \right)} \right).\end{equation*}$$

When we measured the MSD for linear DNA molecules, we noticed that the MSD increased with contour length as expected for a flexible polymer, and that a persistence length of 60 nm fit the data. However, measurements of the MSD for DNA molecules in the intermediate folding states did not follow the fit for a flexible polymer with a persistence length of 10 nm. Instead, the data deviated from the fit at contour lengths >40 nm where the MSD began to decrease. This decrease in MSD would be expected for measurements of the MSD along a circle with a radius of 18 nm.

Finally, we measured the radius of curvature of the DNA (*L* = 105 nm) for (i) linear molecules without protamine and (ii) molecules in the intermediate folding state at 0.2 μM protamine (Figure 6D). We histogrammed the radius of curvature for nearest neighbours along the contour (*ℓ* of ∼4 nm) in both cases. Linear molecules displayed a radius of curvature that had a full width at half maximum (FWHM) of ∼20 nm, ranging from a radius of curvature of ∼5 nm to ∼45 nm. Molecules in the intermediate folding state, on the other hand, had a FWHM of ∼10 nm, with a smaller radius of curvature ranging from <5 to ∼25 nm.

These measurements suggest that the DNA is not being folded entropically by a change in the persistence length. Instead, it seems that the protamine is changing the radius of curvature of the DNA to a value between 5 and 25 nm (∼10 nm). If we assume that every protamine binding site is occupied every ∼11 bp or so (2,14) (producing an 11 bp arc length) and that the radius of curvature is 30 bp, then the DNA curvature we measure would suggest that each protamine molecule is subtending an angle of ∼20° (arc length divided by radius) (Figure 6E).

## DISCUSSION

Here, our goal was to observe the states and dynamics of the DNA looping pathway for protamine. We used a TPM assay to measure real-time folding and an AFM assay to directly image the structures of the DNA. Using these assays, we observed single loops of DNA formed by protamine. These loops form in 105-nm-length DNA molecules and have a measured radius of ∼10 nm. As the 105-nm-length DNA molecules fold into a loop in real time, they enter into multiple folded states that are long-lived (>100 s) and reversible. These folded states are present in short (*L* = 25–50 nm) DNA molecules that are not able to form a loop, indicating that they are intermediate folding states with non-looped structures. Images of the structures of the intermediate folding states showed partial loops of DNA with curved structures, but without an enclosure (*e.g*. c-shapes, s-shapes). These structures have a radius of curvature of ∼10 nm. This dispels the hypothesis that protamine uses a one-step looping model. Instead, protamine loops DNA in multiple steps.

The physical mechanism for folding by protamine is not a decrease in the persistence length of the DNA, but rather a decrease in the enthalpy of the system as the DNA is bent into a loop. A decrease in the persistence length of the DNA creates a more flexible polymer with a higher entropy. Measurements of the shape of the folded molecules showed curved structures that followed similar paths, rather than the random paths expected for a flexible polymer. In addition, the decay of the tangent-tangent correlation was negative instead of levelling off at zero, and the MSD showed decreases at high (>40 nm) contour lengths rather than increases. Thus, the physical mechanism for folding is consistent with an enthalpic decrease in the protamine-DNA system as the protamine bends the DNA into a curve. Assuming full saturation of the protamine on the DNA and a radius of curvature of ∼10 nm, the amount of bending per protamine molecule would be ∼20°. Previous measurements of DNA bending due to positively charged proteins have resulted in 8–17° bends (58), while simulations of bending due to charge neutralization of the DNA produces 6–22° bends (59).

**Figure 7.**
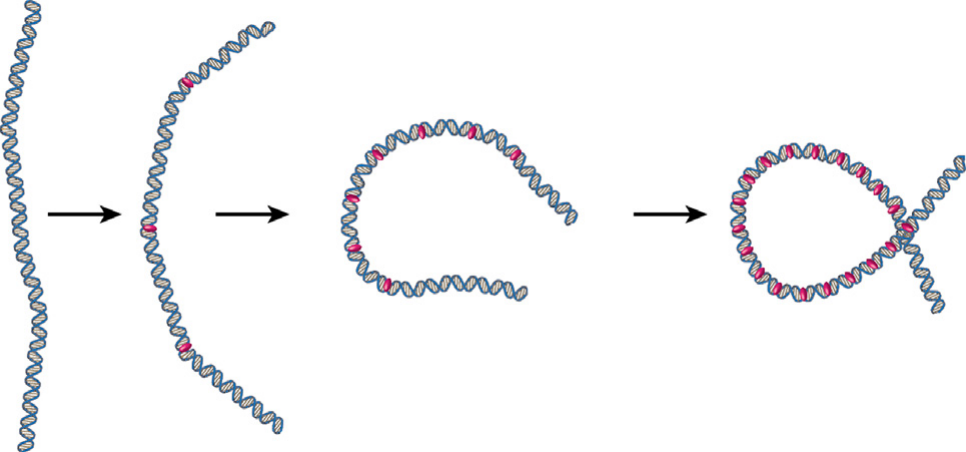
Multi-step looping model. Protamine (*pink*) loops the DNA in multiple steps. Each step is an enthalpic bending of the DNA along a circular path. Multiple steps produce a loop. Bending could be carried out by one or more protamine molecules. Not to scale.

We therefore suggest a model where protamine bends the DNA in multiple steps in order to form a loop (Figure 7). In this multi-step looping model, protamine proteins bind the DNA (either one at a time or in multiples), and cause bending in the DNA. Multiple bending events create the multiple intermediate folding states we observe. Bending could be in-phase, creating c-shapes, or out-of-phase, creating s-shapes. Eventually, enough bending (or some bending and some spontaneous DNA fluctuations) would allow the DNA to cross over itself, creating an opportunity for protamine to interact with both DNA strands and stabilize the loop. We note that this multi-step looping model is in stark contrast to the single-step looping model used by bacterial transcription factors like *lac* repressor (27–29). In a one-step looping model, protamine would have to wait for random fluctuations in the DNA before forming a loop. In this model, protamine can still wait for random fluctuations in the DNA, but it can also bind the entire DNA strand all at once and begin folding, rather than remaining in an unfolded conformation (Figure 1). Thus, this new model of DNA looping seems particularly suited to the function of protamine, which is to condense the entire sperm genome to an almost crystalline packing level (3,4) in minutes (5,60).

One implication of this proposed model is that we might expect longer DNA strands to form more loops and therefore nucleate more toroids. But, observations of protamine condensation on longer DNA strands (60 kbp) show that these long DNA molecules form single toroids (17). To resolve this discrepancy, it may be that the nucleation event for the toroid is not the formation of a single loop. Indeed, previous measurements of DNA unfolding from toroids made by spermine show that toroids are unstable and easily unravel if they contain ≤4 loops of DNA (21).

Another implication is that DNA charge neutralization by protamine has two effects: (i) bending the DNA into a loop and (ii) stabilizing the DNA interactions that form a toroid. Indeed, AFM images of protamine–DNA complexes show both an increased curvature in single molecules due to bending and a propensity for multiple molecules to clump together due to the stabilization of DNA interactions. Since all of our measurements are on protamine bending the DNA into a loop (the first effect) and previous measurements look at protamine stabilizing the DNA into a toroid (the second effect), there is no conflict between our measurements and previous models of toroid formation (Figure 1A). However, future work could test models of toroid formation using longer DNA and the assays we describe here.

We suspect that our looping model for protamine might be generalizable to other biophysical systems. Molecules that form toroids, such as hexaammine-cobalt (III), spermine, and spermidine (19), are likely to use this method. However, other systems that use large populations of positively charged molecules to fold nucleic acids might also use the pathway, including histone-like nucleoid structuring protein (H-NS) (61,62), and histone H1 which forms DNA toroids *in vitro* (63). Still other proteins may use similar pathways, including bacterial nucleoid associated protein HU (64,65) or integration host factor (IHF) (66). Though, there may be some differences. For example, HU has been found to entropically fold the DNA by decreasing the persistence length (64), rather than using the enthalpic folding we see here.

An unknown about the folding of DNA by protamine is about protamine binding and cooperativity. Are protamines binding the DNA individually or cooperatively? Additionally, is folding happening when protamine binds or later on? Maybe folding rather than binding is cooperative? To determine an answer, a real-time folding assay (e.g. TPM, fluorescence-resonance energy transfer, optical tweezers) could be carried out in conjunction with fluorescently labelled protamines to measure binding and folding separately.

Finally, we speculate that folding of DNA by protamine may be useful in nanoengineering DNA structures, including DNA origami (67). Some of the challenges facing DNA origami include the stability, yield, and assembly of large, micron-scale DNA structures (68). Currently, DNA origami structures are stabilized by protein or DNA scaffolds (68) that leave only a few turns of the DNA helix exposed. However, our measurements here show that protamine folds 25-nm-long DNA duplexes, indicating that it might be a good candidate for further stabilization or assembly of DNA origami.

## Supplementary Material

gkaa365_Supplemental_File
