# Supplementary material for: Protamine loops DNA in multiple steps
Source: Nucleic Acids Res. 2020 May 11;48(11):6108–19. doi: 10.1093/nar/gkaa365 (PMC7293030; doi:10.1093/nar/gkaa365)
Supplement: gkaa365_Supplemental_File [file gkaa365_supplemental_file.docx]

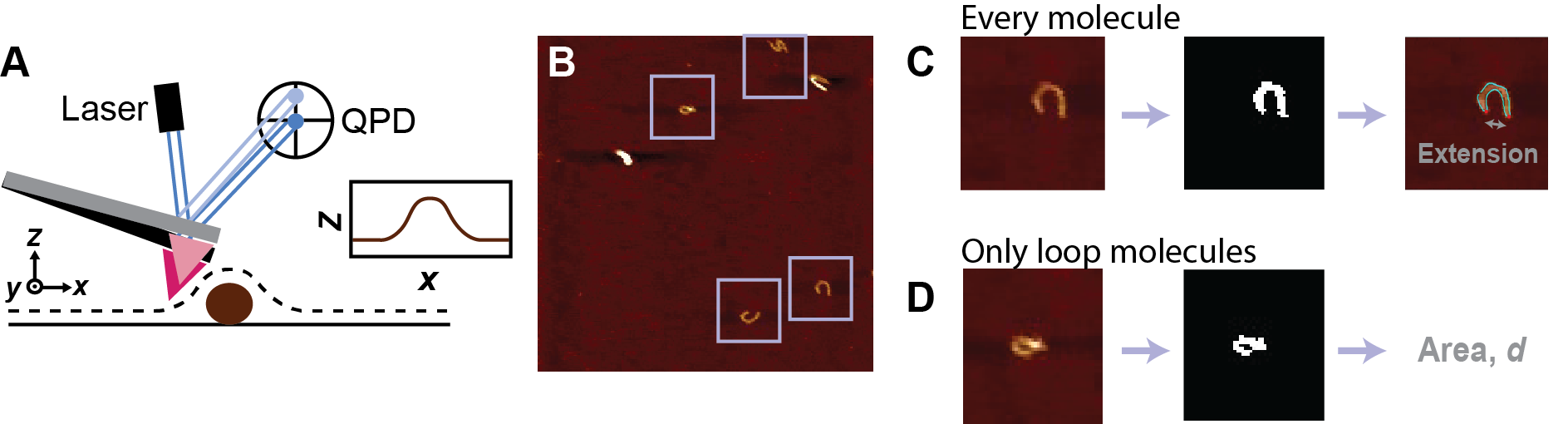


**Figure S1.** **AFM Assay.** A) We adhere DNA molecules (*brown*) to a mica surface (*black solid line*) and scan an AFM in *x* and *y* in air. When the tip encounters a DNA molecule, a laser reflected off the backside of the tip will deflect, causing a change in the location of the laser on a quadrant photodiode (QPD) and a measured movement in *z*. Measurements in *z* as a function of *x* and *y* map out the surface topology. B) An AFM scan of the surface is 1000 nm by 1000 nm and 256 pixels by 256 pixels. Individual molecules in the scan (*purple boxes*) that have a contour length and height within 80% of the nominal value, are separated from all other molecules by ≥1 pixel, and are fully contained within the scan are saved separately as 200 nm by 200 nm images. C) Each individual image is thresholded and the boundary of the DNA molecule (*blue*) and end points (*red*) are extracted using a computer algorithm. The end-to-end distance is saved as the extension of the molecule. D) If the molecule bounds an area, then it is saved as a loop and the bounded area is also extracted. This area, along with the DNA width, is used to calculate the loop diameter, *d*.

**
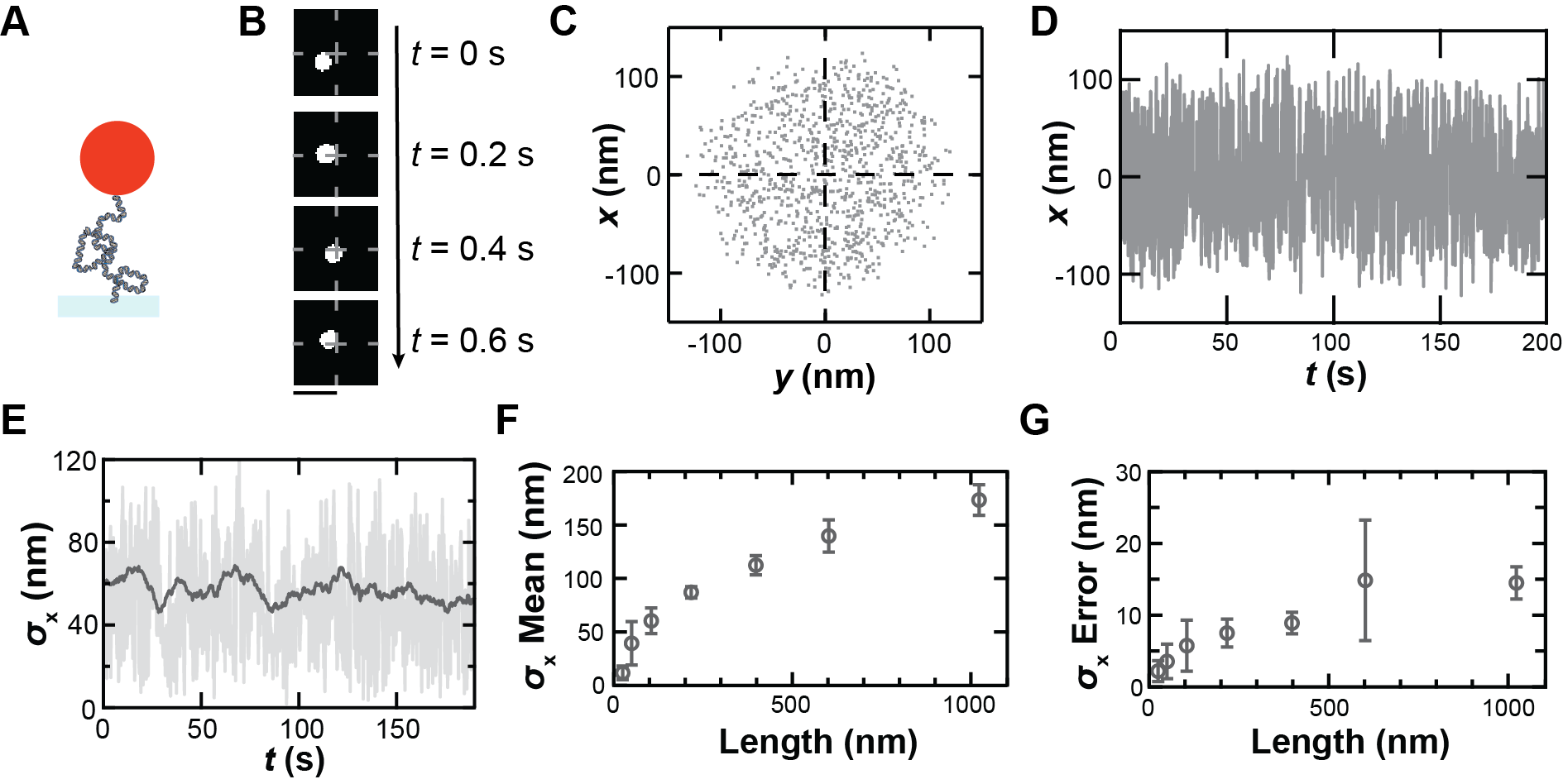
**

**Figure S2.** **TPM Assay.** A) We biochemically tether a polystyrene particle (*red*) to the surface of a coverslip using a DNA molecule. B) We image the tethered particle’s motion over time (*t*) using video microscopy at 5 Hz for 1000 frames. Images are thresholded so that the particle position (*x*, *y*) can be tracked. Scale bar is 1 µm. Crosshairs show the location of the average particle position (0, 0). Length of the DNA molecule is 105 nm. C) Scatter plot of the *y* vs. *x* position of the particle. D) Plot of position in the *x* dimension at 5 Hz for the particle. E) Rolling standard deviation of the *x* trace, *σ*_x_, at 0.1 Hz (*dark*) and 2.5 Hz (*light*) for the particle. *σ*_x_ is calculated by using a rolling window to take the standard deviation of a subset of the *x* trace over time. F) We use the mean of the *σ*_x_ trace (*σ*_x_ Mean) for >20 DNA tethers at each DNA length to create a calibration curve of *σ*_x_ to DNA length. G) We use the standard deviation of the *σ*_x_ trace (*σ*_x_ Error) for >20 DNA tethers at each DNA length as a measure of the error inherent in the assay. The error in the *σ*_x_ measurement is <5 nm for DNA lengths of 105 nm or less.


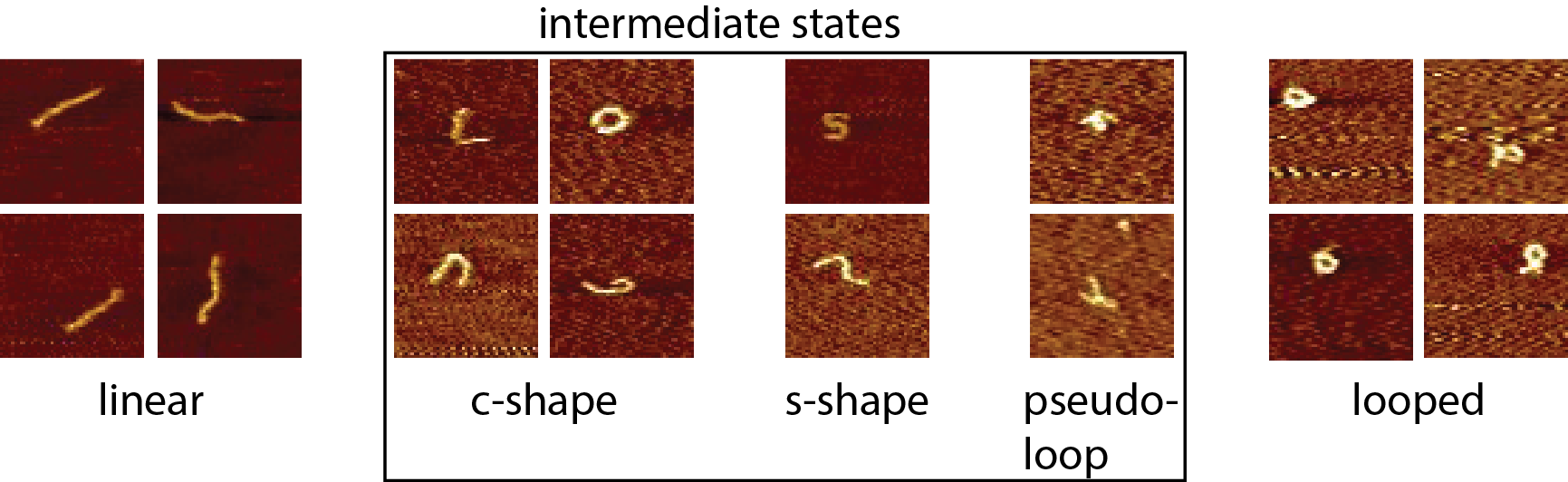


**Figure S3. Classifying 105-nm singlets.** Singlets are classified as linear, looped, or in an intermediate folding state. Linear molecules have a fractional extension >0.6. Molecules in an intermediate folding state have a fractional extension <0.6. Within this category, singlets are additionally classified as c-shape where the curvature is always the same sign (78%), s-shape where the curvature switches signs (20%), or pseudo-loop where the molecule crosses over itself (2%). Looped molecules bound some area.


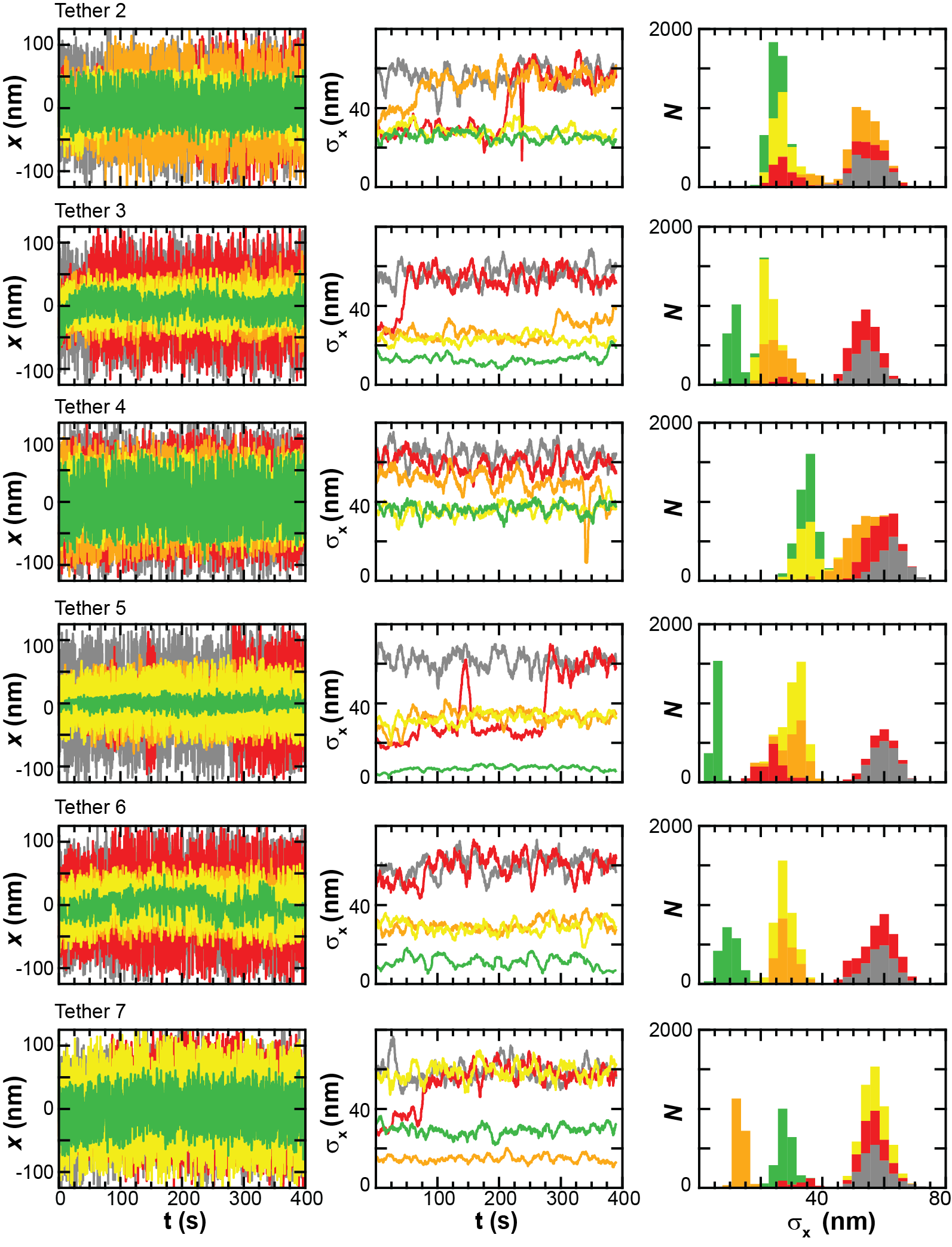


**Figure S4. TPM measurements for 6 additional 105-nm-length DNA tethers.** For each DNA tether, 2-7, we plot the particle position, *x*, at 5 Hz and the rolling standard deviation, *σ*_x_, at 0.1 Hz for each concentration over time. Color scheme same as Figure 2 in the main text. We also plot the *σ*_x_ histogram for the tether at each concentration. Histograms are stacked.


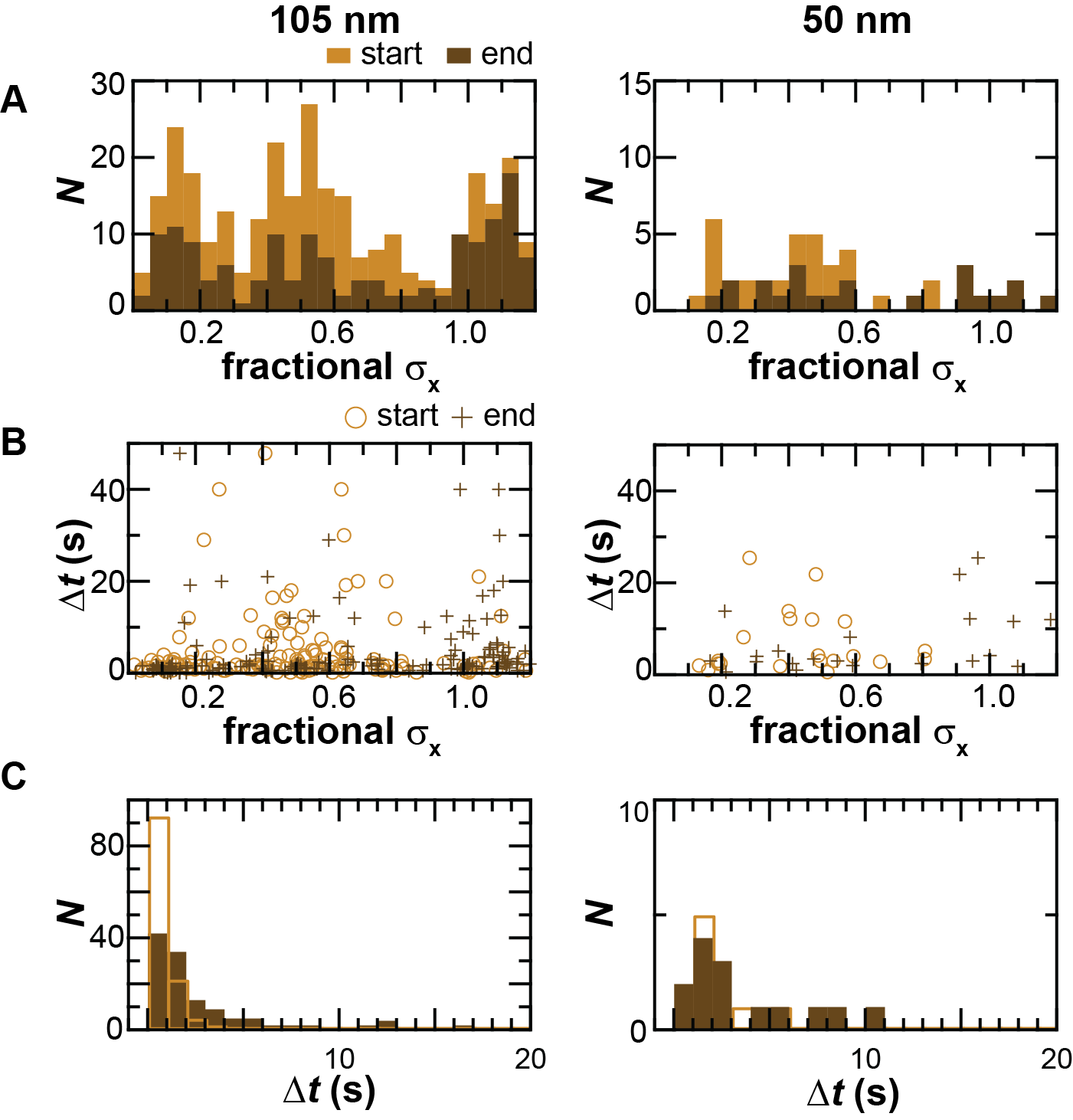


**Figure S5. Characteristics of the transitions for different length DNA molecules.** Transitions are defined to be movements from one peak in the *σ*_x_ histogram to another in the same trace. A) The standard deviation of the particle, *σ*_x_, is recorded at the start and end of each transition and is then divided by the average *σ*_x_ recorded for the tether without protamine, giving the fractional *σ*_x_. This normalization removes the variation in initial *σ*_x_ values. We plot a histogram of the fractional *σ*_x_ for the start (*light brown*) and end (*dark brown*) locations. We record 155 transitions from 105-nm-length tethers (*left*) and 22 from 50-nm-length tethers (*right*). The plots show that transitions are recorded from a range of fractional *σ*_x_ locations, with a peak at 1.0 for the unfolded state and <1.0 for the folded states. B) The transition time, *Δt*, or the time it takes to transition from the start *σ*_x_ to the end *σ*_x_ is determined from a rolling standard deviation trace with a window of 2 frames. This trace at 2.5 Hz can measure transitions longer than 0.4 s. The transition time is plotted for each start (*light brown circle*) and end (*dark brown cross*) location. Longer transition times (>2 s) are mostly reversals which show the start location at a smaller fractional *σ*_x_ than the end location. C) A histogram of the transition time shows that most transitions (43 out of 56 forward transitions and 50 out of 99 reversals for *L* = 105 nm, 5 out of 7 forward transitions and 9 out of 15 reversals for *L* = 50 nm) have transition times that are ≤2 s, indicating a discrete transition. However, about half of the reversals show transition times that are >2 s.


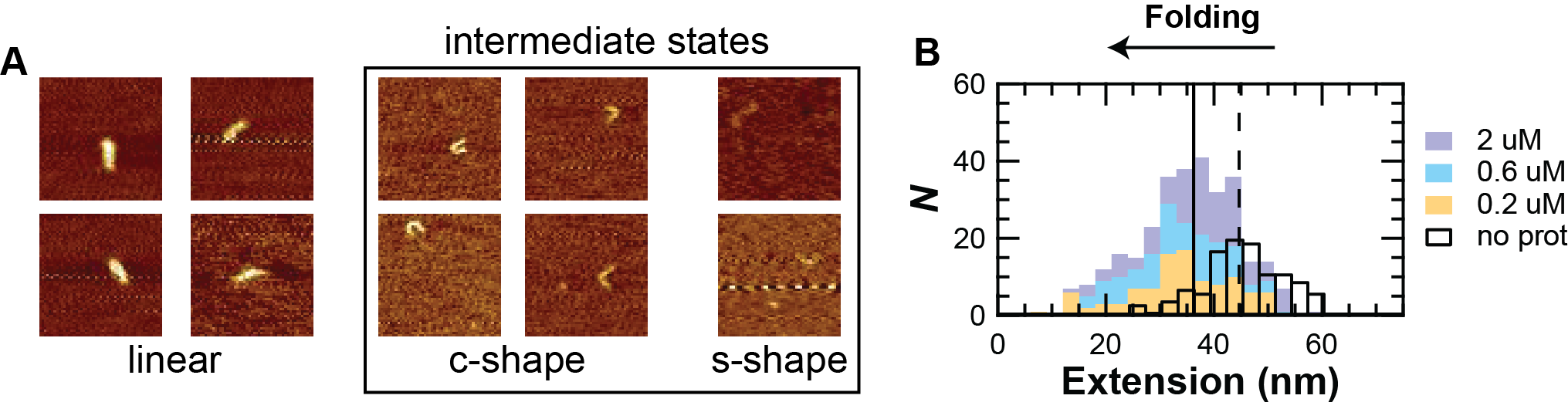


**Figure S6. 50-nm length DNA ­­­folds into intermediate structures.** A) Molecules are classified as either linear or in an intermediate folding state. Molecules in the intermediate folding state could be c-shapes (96%) or s-shapes (4%). The molecules do not loop. B) Stacked histograms of the extension for 50-nm-length DNA without protamine (*black*) and with 0.2 µM (*orange*), 0.6 µM (*blue*), or 2 µM (*purple*) protamine, indicate that the DNA is folding. Unfolded molecules have a mean extension of 45 ± 7 nm (*dashed line*), while molecules with protamine show a decrease in the extension to a mean value of 35 ± 9 nm (*solid line*). From the histogram we can also see that without protamine there are 4 out of 115 molecules (3%) that have extensions <30 nm (fractional extension <0.6). With protamine, the number jumps to 82 out of 301 molecules (27%). We label molecules with extensions <30 nm as molecules in the intermediate folding state.

| **DNA Length (nm)** | **[Protamine] (μM)** | **Number of Singlets** | **Number of Loops** |
| --- | --- | --- | --- |
| 50 | 0 | 114 | 0 |
|  | 0.2 | 101 | 0 |
|  | 0.6 | 87 | 0 |
|  | 2 | 113 | 0 |
| 105 | 0 | 95 | 4 |
|  | 0.2 | 94 | 20 |
|  | 0.6 | 88 | 31 |
|  | 2 | 121 | 34 |
| 217 | 0 | 103 | 4 |
|  | 0.2 | 92 | 13 |
|  | 0.6 | 94 | 38 |
|  | 2 | 96 | 62 |

**Table S1. AFM image counts for different DNA lengths and protamine concentrations.**
